# Wireless Sensor Node for Surface Seawater Density Measurements

**DOI:** 10.3390/s120302954

**Published:** 2012-03-02

**Authors:** Federico Baronti, Gabriele Fantechi, Roberto Roncella, Roberto Saletti

**Affiliations:** Dipartimento di Ingegneria dell’Informazione: Elettronica, Informatica, Telecomunicazioni, University of Pisa, Via Caruso 16, Pisa 56122, Italy; E-Mails: f.baronti@iet.unipi.it (F.B.); g.fantechi@iet.unipi.it (G.F.); r.roncella@iet.unipi.it (R.R.)

**Keywords:** wireless sensor network, seawater density measurement, magnetostrictive displacement sensor

## Abstract

An electronic meter to measure surface seawater density is presented. It is based on the measurement of the difference in displacements of a surface level probe and a weighted float, which according to Archimedes’ law depends on the density of the water. The displacements are simultaneously measured using a high-accuracy magnetostrictive sensor, to which a custom electronic board provides a wireless connection and power supply so that it can become part of a wireless sensor network. The electronics are designed so that different kinds of wireless networks can be used, by simply changing the wireless module and the relevant firmware of the microcontroller. Lastly, laboratory and at-sea tests are presented and discussed in order to highlight the functionality and the performance of a prototype of the wireless density meter node in a Bluetooth radio network. The experimental results show a good agreement of the values of the calculated density compared to reference hydrometer readings.

## 1. Introduction

Monitoring the aquatic environment is becoming increasingly important, particularly in terms of pollution detection, marine surveillance and seawater data collection. In fact, environmental awareness throughout the World has led to the need for applications for exploring, understanding, and protecting the aquatic environment. The new technologies give an important contribute to the improvement in techniques, procedures and equipment applied to the collection of data from the aquatic environment [1].

Besides the observations performed using airborne or satellite instrumentation, one of the most promising techniques for collecting marine environment data is to deploy wireless sensor networks (WSNs), in which every node of the network is capable of locally collecting particular data by means of its own sensors, data that are then exchanged and transmitted over the network to remote users. Many research groups have therefore developed architectures, protocols, hardware, sensors, management policies in order to efficiently set-up and operate WSNs in marine environments. These networks are usually located and operated underwater, but surface nodes may also be included [2–4].

There are many possible parameters or information that each node in the sensor network can collect. Among others, water parameters such as temperature, salinity, conductivity, turbidity are important to determine the status of the point where the measurement is performed. The collection of water parameters at many points using a sensor network enables 3D profiles of the water characteristics to be reconstructed. One useful parameter in understanding the properties of seawater is density. Density is the fundamental parameter that governs floatation in a fluid and is thus important both for scientific purposes and for applications where accurate control of buoyancy is mandatory.

Seawater density is usually measured indirectly. In fact, knowledge of water salinity values, together with temperature and pressure allows us to derive the density. Great effort is being made in improving the algorithms that lead to an estimation of the density performed in this indirect way. There are many tables or computer programs by which density values can be derived by the knowledge of salinity, temperature and pressure [5,6]. For instance, a 25-term polynomial is proposed in [7] to fit the experimental findings. Recent observations have extended the range of temperature and absolute salinity in which the equation of state of seawater is valid [8].

Seawater density can also be measured directly. Many procedures or techniques can be used to measure the density of a liquid, according to the definition of density as mass over volume of a given sample [9,10]. Typical instruments for measuring water density include the hydrometer [11] and the pycnometer, by which the density of a liquid relative to a reference (usually distilled water) is measured. However, if we want to use them in extended and thorough observations of the marine environment, these measurement principles and the corresponding instruments need to be suitable for a sensor network, and not only for laboratory experiments. Therefore, sensors are needed with dedicated electronic interfaces, in order to easily measure the density and be part of a sensor network. The density of a liquid can be measured from the resonance of a vibrating element put in contact with the sample, the frequency of which is dependent on the liquid density itself [12,13]. Density can also be measured using ultrasounds, by determining the reflection coefficient of the ultrasound at the interface between the liquid and a quartz glass [14].

There are two density measurement principles that seem very suitable for implementation in a node of a WSN because of their simple application. The first is based on measuring the difference in hydrostatic pressure between two vertically aligned points below water level, which depends on the water density. The second is based on measuring the buoyancy force of a floating object, which depends on the liquid density according to the Archimedes’ law. This force is known if the volume of the immersed part of the object is also known. The density calculation is related to the measurement of the immersion of a floating object and is thus reduced to the measurement of a linear displacement [11,15,16].

The aim of this paper is to extend the findings mentioned above and to present the design of a seawater electronic density meter based on the buoyancy force measurement carried out using a magnetostrictive linear displacement sensor. A preliminary description of this meter, used in a WSN for measuring the freeboard of cruising yachts, can be found in [17]. We extend here the brief description in [17] by recalling the theory of operation, presenting in detail the design of the prototype and showing some new experimental results that demonstrate its functionality and characterize its performance. The laboratory experiments were carried out by equipping the sensor with a radiofrequency (RF) standard wireless interface (namely Bluetooth). Our particular design means that other kinds of wireless interfaces could also be used, either RF or ultrasonic based, to enable the meter to be inserted into both surface and underwater WSNs.

## 2. Density Meter Operation Principles

The two seawater density measurement principles mentioned above (hydrostatic pressure and buoyancy force measurement) differ in many aspects. First of all, the physical quantity to be measured is profoundly different. The density of a liquid can be calculated by measuring the difference of the hydrostatic pressure in two vertically-aligned different points of the liquid. The idea was originally applied to the measurement of the distance between the two probe points (*i.e.*, the liquid level) [18], but it also applies to the density measurement of the liquid, assuming the vertical distance between the two points is known. Water pressure is the physical quantity to be measured in this case. The second approach relies on the measurement of the buoyancy force of a floating object, the immersion of which depends on the liquid density, according to the Archimedes’ law. The vertical displacement of the float is the physical quantity to be measured in the second case. It thus should be noted that the first method is applicable at different depths of water, whereas the second is only useful for measurements at the sea surface since it is applied to a floating object. However, the second method seems very appealing, because a very high resolution and accuracy of linear displacement measurements is achieved if electronic linear displacement sensors are used. Therefore, a high accuracy is also expected in the density measurement. Magnetostrictive (MS) linear displacement sensors [19,20] are transducers that read a displacement with high-accuracy and reliability, thus matching the requirements of harsh and hostile environments, since these transducers are robust and waterproof. They are excellent candidates for the design of a seawater density meter according to the principle that will be described later; they are also affordable [21,22]. Unfortunately, none of the sensors available on the market are equipped with wireless interfaces.

The operation principle of an MS sensor is based on the measurement of the time of flight of a pulse along a metal ferromagnetic rod, in which a permanent magnet probe positioned along the rod determines a local micro-twisting due to the interaction with the ferromagnetic material. The pulse starts as an electric pulse that is transformed in a backward-moving ultrasonic pulse at the point where the electric pulse encounters the micro-twisting. The pulse time of flight measurement enables the calculation of the distance of the magnet (our measurement probe) from the source. If more than one magnet is placed along the sensor rod, the electric pulse determines the onset of a number of ultrasonic pulses, one for each position magnet. It is thus possible to use the same sensor to measure the displacements of many magnet probes placed along the measuring rod.

The basic idea behind the wireless density sensor node described here was first introduced in [11], where the principles of a classical hydrometer were applied to an electronic circuit. A weighted float suspended in a container where the liquid is maintained at a given height will be less immersed if the liquid is denser, according to Archimedes’ law. The authors measured the weighted float immersion with a Linear Variable Differential Transformer (LVDT) and derived the liquid density. A commercial instrument based on the same principle exploits a permanent magnet in the float and measures the float immersion with Hall sensors [16]. In [15], the authors used the MS linear displacement sensor as a measuring device for the displacement, and used two magnet probes to sense the liquid surface level and the immersion of the weighted float. However, the device was not designed to be connected to a network, either wired or wireless. Our wireless density sensor network node uses the same operating principles, but it has a wireless interface to enable use in a sensor network.

**Figure 1.**
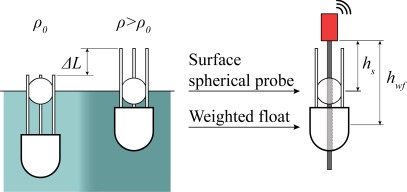
Operating principle of the density meter. A spherical probe floats at the water surface, while a semi-floating probe is partly immersed. The immersion depends on the water density. The MS sensor accurately measures the displacement difference between the two probes, from which the density is calculated.

Let us briefly recall the theory behind the functionality of the hydrometer and how it is used with this electronic instrument. Figure 1 shows the working principle of the density meter. The MS sensor has two magnet probes and its rod is immersed in the seawater. The first probe is located inside a sphere that floats at the sea surface. The second probe is inserted in another structure, the weight of which is chosen so that the structure is semi-floating below the first sphere. If we assume that the floating sphere immersion is constant with the water density, this probe gives a displacement measurement *h_s_* that corresponds to the surface level of the liquid. Instead, the second probe in the semi-floating structure is submerged more or less according to the water density. Therefore, its displacement *h_wf_*, as measured by the MS sensor, changes according to the liquid density. The difference between the two readings gives the exact immersion of the semi-floating structure and is thus related to the buoyancy force and the water density value.

If we define ρ_0_ as the water density and *V*_0_ the corresponding immersed volume of the semi-floating structure, this structure is subjected to a buoyancy force *F_b_* that is equal to the gravity force, according to Archimedes’ law:

(1) $$F_{b}=\rho_{0}gV_{0}$$

where *g* is the acceleration of gravity. If the water density is changed to a new value ρ, the buoyancy force tends to change and the structure draft changes accordingly, until a new equilibrium of the gravity and buoyancy forces is reached, with a different immersed volume of the structure. The top part of the semi-floating structure has a tripod shape, made up of three cylindrical rods. The immersed volume changes to *V*, which is only due to a shift Δ*L* in the immersion of the three cylindrical rods of radius *r*. Therefore, we can write:

(2) $$F_{b}=\rho \mathrm{gV}=\rho g(V_{0}+\Delta L\cdot 3{\pi r}^{2})$$

and thus:

(3) $$\rho_{0}gV_{0}=\rho g(V_{0}+\Delta L\cdot 3{\pi r}^{2})$$

We finally obtain:

(4) $$\rho =\frac{\rho_{0}}{1+\frac{\Delta L\cdot 3{\pi r}^{2}}{V_{0}}}$$

Equation (4) shows that the density value is a function of the displacement variations between the two probes, with reference to a calibration point where the values of ρ_0_ and *V*_0_ are known, as happens in a classical hydrometer.

One of the advantages of this solution is the differential measurement, because the water surface level and the draft of the semi-floating structure are measured simultaneously, without controlling the liquid level as it is required in [11]. As a consequence, the density meter can operate in an uncontrolled floating housing, because the reference water surface level is always measured by the instrument itself.

## 3. Density Meter Design

Commercially available liquid level meters based on MS linear displacement sensors usually have different types of wired interfaces (analog, CAN bus, Profibus, Ethernet, SSI, *etc*.), to be used in industrial environments. A MS sensor suitable for the density meter must be able to measure the displacements of two magnetic probes, in order to obtain the measurement of Δ*L*, which leads to the calculation of the density according to Equation (4). Therefore, the liquid level sensor needs a second magnetic probe inserted in a semi-floating structure, the physical dimensions of which have to be known very carefully to provide the *r* and *V*_0_ values of Equation (4). In addition, the system needs to be equipped with a wireless interface and an autonomous power supply, which allow the insertion as a node of a sensor network.

### 3.1. Density Meter Physical Design

As first design step of the density meter we need to choose the design parameters of the sensor unit, according to the operating principle detailed above. The dynamic range of the density measurement we want to achieve determines the minimum and maximum values of Δ*L* and thus the sensor measurement range. Other design parameters are set by the expected resolution and accuracy that need to be achieved.

Let us define the relative density variation Δρ/ρ_0_ referring to the reference point ρ_0_ as:

(5) $$\frac{\Delta \rho }{\rho_{0}}=\frac{\rho -\rho_{0}}{\rho_{0}}=\frac{\frac{\rho_{0}}{1+\frac{\Delta L\cdot 3\pi r^{2}}{V_{0}}}-\rho_{0}}{\rho_{0}}=-\frac{\Delta L\cdot 3\pi r^{2}}{V_{0}+\Delta L\cdot 3\pi r^{2}}$$

and the variation of the immersed volume of the structure Δ*V* = Δ*L* · 3*πr*^2^. From Equation (5) we can write:

(6) $$\frac{\Delta V}{V_{0}}=-\frac{\frac{\Delta \rho }{\rho_{0}}}{1+\frac{\Delta \rho }{\rho_{0}}}$$

Therefore, the relative variation in density can be approximated with the relative variation in the immersed volume, if we neglect Δρ/ρ_0_ with respect to the unity. This means that the immersed volume should change a factor 10^−3^ to measure a 10^−3^ relative change in density.

The weighted float was thus designed by us with the shape shown in Figure 1 and built with the following physical parameters: immersed volume *V*_0_ = 57 × 10^4^ mm^3^, radius of the tripod rods *r* = 3 mm, radius of the floating sphere R = 30 mm. We can derive the expected dynamic range of Δ*L* for a given interval of densities from Equation (6). Thus, we need an MS sensor with a dynamic range of 300 mm, if we want to measure density in the range 1,000–1,050 kg/m^3^. If we also consider the waves that cause dynamic fluctuations in the sensor probes and thus the measurements, we need a MS sensor with a stroke length longer than 300 mm, to allow the probes to move freely along the rod. We chose a commercial MS sensor with a stroke length of 600 mm for our prototype. The sensor is a liquid level measurement system commonly used in industrial environments [22]. Unfortunately, commercial liquid level MS sensors are not equipped with semi-floating probes, so that we customized the sensor by adding the above described semi-floating structure to it.

Equation (5) is also useful to calculate the maximum resolution achievable when the physical parameters of the float are known. The sensor adopted in our density meter prototype has a resolution in the linear displacement measurement of around 50 μm, so that this value is also the resolution of the Δ*L* value. If we use this value in Equation (5) as the minimum detectable displacement variation Δ*L*, we achieve a minimum theoretical resolution of the density value equal to 7.44 × 10^−6^. This value is obviously a theoretical bound given by the digital nature of the displacement measurement and is not achievable because of the many factors for which the accuracy obtainable is worse than resolution. If we consider the absolute accuracy of the displacement reading to be of the order of 1 mm [17] and the same value as the accuracy of a differential measurement, then from Equation (5) the accuracy achievable with the density sensor is 1.49 × 10^−4^ (*i.e.*, about 150 ppm). The above calculated values make the meter very appealing for monitoring sea environments and are better than those reported for a commercial stand-alone similar instrument [16].

### 3.2. Wireless Node Electronic Design

**Figure 2.**
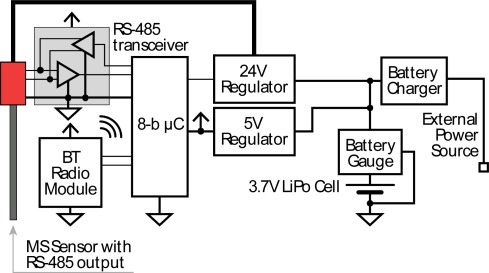
Architecture of the electronic part of the sensor node. The wireless module can be substituted with other types of modules by simply changing the microcontroller firmware.

The MS linear displacement sensor provides a digital reading of the displacements of the two cursors on an output interface, in our case an RS-485 industrial interface. We need to provide additional electronics for its insertion as a node in the WSN. The sensor needs a wireless connectivity and a power supply source, to operate as an autonomous node. The wireless technology to be used strongly depends on the network in which we intend to insert the sensor node. The network could be optical, acoustic, or radiofrequency (RF) based, depending on the application [23–25]. In any case, the architecture chosen enables us to easily change the wireless link type and adapt the node to different networks. In fact, the wireless module can easily be replaced with other types, because the communication between the wireless module and the sensor is not direct. Instead, it is created by means of a 8-b microcontroller (μC) that acts as a bridge between the sensor and the wireless module, so that a different module type is managed by only changing the communication firmware. Figure 2 shows the architecture of the electronic part of the sensor node.

The proof-of-concept demonstrator uses a Bluetooth (BT) radio as wireless module. We chose BT because our aim was to show the feasibility of the density meter node, rather than characterizing it on a particular application in the field. BT makes control of the density meter node very easy, because the master node of the network can be a personal computer (PC) located at distance less than 100 m. However, the BT radio module could be substituted with a long-range radio transmitter module or an underwater ultrasonic transmitter module, should the density meter be used in a wide-area sensor network. The BT radio is provided with a 5 V USART interface directly connected to the μC. Instead, a RS-485-to-USART transceiver is needed for communication between the MS sensor and the μC.

The wireless density meter node is supplied with a Lithium-Polymers (LiPo) cell, the voltage of which is 4.2 V when fully charged and 3 V when discharged. The capacity of the battery cell determines the battery duration and thus the operational time of the wireless density meter node, before the battery needs to be recharged. The most energy-consuming component of the density meter is the MS sensor that requires a 24 V power supply and consumes about 2 W when carrying out a displacement measurement. The maximum continuous measuring time *t_meas_* can be written, neglecting all the other power consumptions, as:

(7) $$t_{\mathrm{meas}}=\frac{V_{\mathrm{cell}}C_{\mathrm{cell}}}{P_{\mathrm{MS}}}$$

where *V_cell_* is the cell voltage, *C_cell_* is the cell capacity (expressed in Ah) and *P_MS_* is the power needed by the MS sensor. We used a small 3.3 Ah LiPo cell in the prototype. The continuous measuring time is thus 6 h, which is not long enough for a successful application as a wireless sensor node. However, the operating time of the node can be significantly increased by adopting an effective energy saving policy. In fact, if we consider that the water density variations are very slow over time, continuous operation of the node is meaningless, because measurements taken every 3–6 h are sufficient. Let us consider 120 s as a time sufficient to acquire enough displacement data and to obtain a reliable measure of the water density by averaging the waves and disturbance components. The MS sensor can thus be powered only when needed, in order to reduce the working duty cycle and the average power consumption. If the sensor is turned on for 2 min every 3 h by the μC, which periodically enables the 24 V voltage regulator supplying the sensor, the average power consumption is about 22 mW. The MS sensor average power consumption $\bar{P_{\mathrm{MS}}}$ is now comparable with the average power consumption of the radio module $\bar{P_{\mathrm{radio}}}$ and of the other electronic devices $\bar{P_{\mathrm{standby}}}$, so that Equation (7) is rewritten as:

(8) $$t_{\mathrm{meas}}=\frac{V_{\mathrm{cell}}\;C_{\mathrm{cell}}}{\bar{P_{\mathrm{MS}}}+\bar{P_{\mathrm{radio}}}+\bar{P_{\mathrm{standby}}}}$$

$\bar{P_{\mathrm{radio}}}$ can be calculated by multiplying the rated power consumption of the radio module by its operating duty cycle. When the the μC turns on the radio module, it takes very little time (fractions of a second) to transmit the measurement data. However, the radio module is turned on more often than the MS sensor because of the communication tasks needed in order to be part of the WSN. This is when the node sends diagnostic data and receives data and/or commands from the other nodes, in accordance with the protocol adopted in the network. A reasonable estimation of the $\bar{P_{\mathrm{radio}}}$ value in our prototype is around 5 mW. Instead, $\bar{P_{\mathrm{standby}}}$ takes into account the average power consumption of all the other electronic components of the wireless node. Some can be operated in low-power mode by the μC, to save energy when idle. This part of the consumed power is estimated to be around 3 mW. As a consequence, the battery duration is extended to more than 400 h, with the small size battery we used in the prototype. This time can be further extended by reducing the frequency of the measurements or by using a cell with a higher capacity. In any case, there is a trade-off between the operational time of the node and the size and cost of the Li-Po battery.

The node electronics also recharge the battery, by means of the integrated battery charger shown in Figure 2, which is connected to an external 5 V power source, when needed. Our prototype density meter is recharged through a USB port or a wall adapter but the external 5 V power source could also be provided by the regulated output of a solar panel, in open sea unattended operations.

## 4. Error Sources

Several factors can introduce errors that make the above assumptions inexact, although still acceptable. As an example, the assumption that considers the immersion of the floating probe, which measures the water surface level, constant is not true. In fact, the floating probe immersion changes slightly with the water density and an error is introduced in the displacement measurement. However, the error can be compensated for by applying a correction factor to the reading. The factor is calculated by taking the floating probe shape (a sphere in our case) into account, as described in [15].

Temperature is a crucial factor for the accuracy of the reading, as the propagation speed of the ultrasonic pulse in the sensor rod is strongly affected by the temperature. If we provide the MS sensor with a temperature probe, we can apply a compensation algorithm that takes into account the ultrasonic speed variation with the temperature and correct the displacement measurement accordingly. A factor 9 improvement in the absolute accuracy of a linear displacement MS sensor was obtained with the compensation of the temperature in [17]. However, both readings with which the difference is calculated, are affected by temperature in the same way. Therefore, the errors made in each measurement tend to cancel each other.

Another error source is the hysteresis in the equilibrium position of the magnetic probe for the displacement measurement. The floating sphere and the weighted float move along the MS sensor rod and are subjected to a friction force that should be taken into account in Equation (1). The friction force determines an equilibrium point that may be different from test to test in experiments repeated in identical conditions. This difference leads to an equilibrium interval instead of an equilibrium point and thus an error in the measurement. The amplitude of this interval is the measurement hysteresis, which can be minimized by reducing the friction as much as possible. Some experimental results regarding hysteresis measurements are reported in the following section.

Finally, there are possible error contributions due to the dependence of the density on the water temperature. The water temperature can be easily measured by the density sensor, by providing it with a temperature probe. Knowledge of the water temperature value enables the density to be calculated correctly even at different temperatures from the reference point, where the density meter was calibrated.

## 5. Experimental Results and Discussion

**Figure 3.**
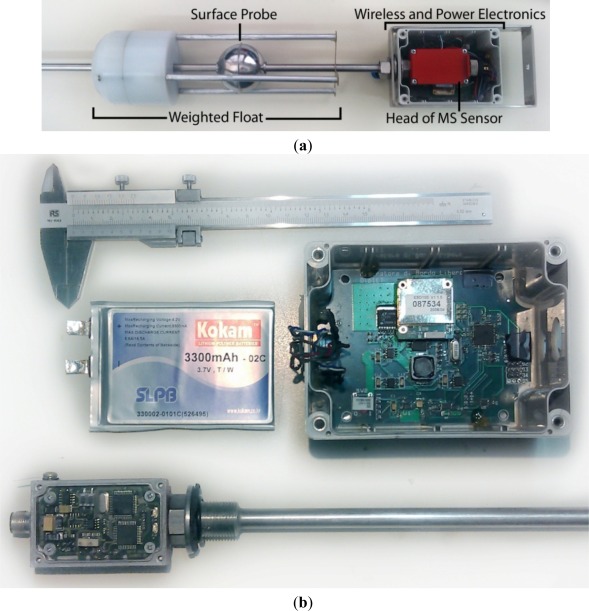
(**a**) Photograph of the complete wireless density meter node. Note the sensor rod with the two probes: the surface floating sphere and the tripod weighted float; (**b**) Density meter electronics consist of the Li-Po battery, the custom wireless board and the MS sensor board, part of the commercial MS liquid level meter customized here.

A proof-of-concept prototype of the wireless density meter node was designed and tested by means of laboratory and at-sea tests. Figure 3(a) shows a photograph of the complete structure, where the sea surface probe (sphere), the weighted float (tripod) and the electronics board of the wireless node are shown. The density meter node is shown disassembled in Figure 3(b). The custom board that provides the MS sensor with the wireless connectivity and the power supply together with the LiPo Battery are visible.

The laboratory experimental set-up consists of a tank filled with distilled water, in which a controlled quantity of salt can be added to increase the water density. The rest of the wireless network is emulated by a PC running a LabVIEW program that establishes the wireless connection with the node via the Bluetooth link, sends commands to the node to start the acquisition and receives back the results of the measurements. The firmware running on the node μC receives the start command from the PC, turns on the MS sensor, receives the measurement results on the RS-485 interface, translates and packs them in a data structure which is sent back to the PC on the Bluetooth link. When the measurement is finished, the μC firmware turns off the sensor and activates a low-power consumption mode, from which it is awoken periodically, to save energy. Finally, the results of the measurements are displayed on the PC screen.

**Figure 4.**
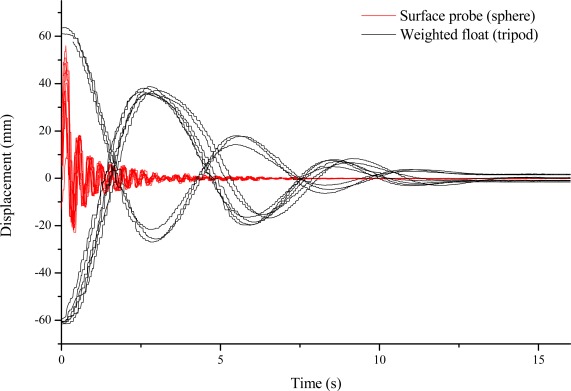
Transient responses of the displacement readings of the two probes, when subjected in laboratory to repeated forced immersions and subsequent release. Note the hysteresis in the equilibrium points of the tripod weighted float.

First of all, the density meter was calibrated in a laboratory by comparing the measurements carried out in the distilled water tank at room temperature with the reading of a glass hydrometer for the same water sample. No compensation in temperature and sphere immersion was applied. Other experiments were then carried out to test the density meter and to characterize its behavior. Both the sea surface probe and the weighted float were forcedly immersed and then released. The displacement reading was acquired as a function of time, as reported in Figure 4, to show the dynamic response of the two probes when taken out of the equilibrium. The experiment shows repetitive damped oscillatory responses that are quite different in the two cases, particularly in terms of the time constants. The sphere is quicker in response, so that it can follow the water surface oscillations, whereas the immersed probe is slower and thus less sensitive to oscillations.

These experiments were repeated several times to show the presence of hysteresis in the measurements. In fact, the regime value of the displacement is reached when the structure reaches the equilibrium point between the gravity and buoyancy forces. Unfortunately, this point varies because of the friction forces that are applied to the structure when moving along the sensor rod. This is confirmed by the experimental results, which show different regime values in the displacement after repeated forced immersions. These values define a hysteresis range of about 3 mm. Instead, the floating sphere does not show hysteresis, because of the very loose contact with the rod. However, this is a case when laboratory experiments produce worse results than in the field. In fact, the unavoidable movements of the sea water layer where the density meter is placed act as a noise source that is superimposed on the probe displacement and tends to average the hysteresis effect and compensate for it.

**Figure 5.**
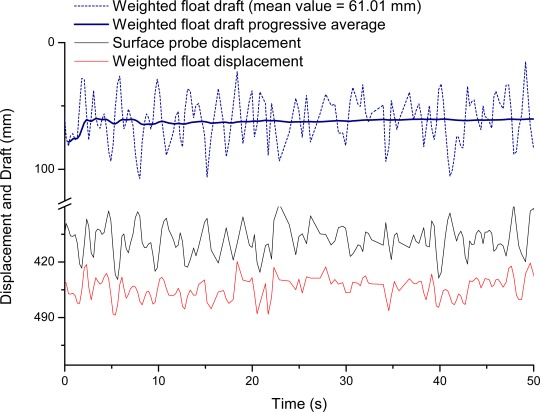
Displacement readings of the two probes in at-sea test experiments. Note the large oscillations due to the waves and the differential reading that gives the weighted float draft. The solid line shows the progressive average of the draft that filters out the effect of the oscillations after just few seconds of measurement.

The density meter node was also tested at sea, by hanging it from a pier in a marina. Several acquisitions were performed. An example of the results obtained is reported in Figure 5, which shows the position of the probes along the sensor rod referring to the sensor head position. It is easy to see how these displacements are affected by strong oscillations due to the sea waves. This condition is the worst possible, because the sensor is placed on a fixed pier and the displacement measurements are fully affected by the wave dynamics. Should we repeat the measurements with the sensor placed in a floating container, we would experience a smaller influence of the waves on the displacement measurements, because both the sensor head and the probes are moving with the waves. Therefore, we could expect more stable and less sensitive results, particularly in the difference between the displacement values of the two probes.

Instead, a floating container may determine possible deviations from the verticality of the sensor, which affect the accuracy of the measurement. Figure 5 also reports the displacement difference of the probes (*i.e.*, the weighted float draft), which leads to the calculation of the water density, and the progressive average value displayed as a function of time. It is worth noting how the large fluctuations are averaged and that the mean value reaches a rather constant value after just few seconds of measurement. The density value calculated according to Equation (4) from the experiment described above was 1,015 kg/m^3^, in good agreement with the value 1,014 kg/m^3^ measured with the glass hydrometer.

**Figure 6.**
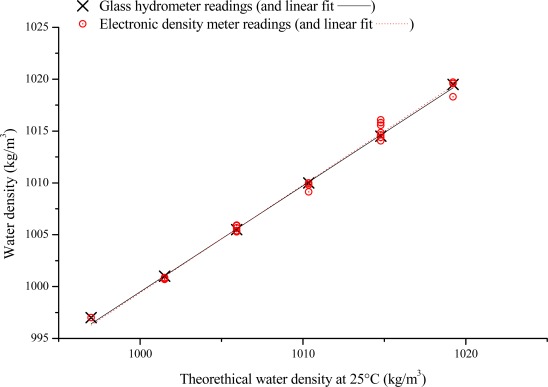
Water density values calculated by the wireless density meter node and by the reference glass hydrometer, as a function of the theoretical density value determined by the quantity of diluted salt in distilled water.

Finally, laboratory tests were also performed at room temperature (25 °C) in water with different density values. A controlled quantity of salt was added to the distilled water and the sensor node was used to calculate the density. The density was also measured at the same time with the reference glass hydrometer. Figure 6 shows the values of the densities measured by the wireless sensor node and by the hydrometer as a function of the theoretical water density value at that temperature, as determined by the amount of salt added to the distilled water. It is worth noting that the density calculated by the wireless sensor node is in excellent agreement with the reference value measured. Finally, the knowledge of the water temperature enables compensation for the temperature induced variations of the water density and calculation of the density at 4 °C for that seawater sample.

## 6. Conclusions

This paper has described the design and the experimental characterization of a density meter based on the use of MS linear displacement sensors suitable for insertion in a wireless sensor network for the observation and monitoring of the seawater environment. The operating principles of the meter, the design of its mechanical and electronic parts and its features have been described, particularly the attainable accuracy that is in the order of hundreds parts per million. The main innovation of the work is the addition of an electronic board based on a microcontroller to an MS commercial sensor, which makes the MS sensor able to be inserted in a wireless sensor network, irrespectively of the type (acoustic, radio or optical). The simple change in the wireless module and the microcontroller firmware means that the node can be easily used in underwater or above surface wireless networks.

The wireless density meter node was also characterized with laboratory and at-sea tests, which proved its full functionality and good response even in the noisy environment caused by sea waves. Both the static and dynamic responses of the sensor have been described and the hysteresis effects measured and discussed. Finally, the values of the density of different water samples were calculated and compared with a reference hydrometer reading, showing a good agreement. In conclusion, the wireless density meter node proposed seems to be an efficient, low-cost and reliable solution for measuring and monitoring density, an important seawater parameter, in marine wireless sensor networks.
